# Factors influencing the uptake of public health interventions delivery by community pharmacists: A systematic review of global evidence

**DOI:** 10.1371/journal.pone.0298713

**Published:** 2024-08-01

**Authors:** Audrey Mumbi, Peter Mugo, Edwine Barasa, Gilbert Abotisem Abiiro, Jacinta Nzinga

**Affiliations:** 1 Health Economics Research Unit (HERU), KEMRI-Wellcome Trust Research Program, Nairobi, Kenya; 2 Nuffield Department of Medicine, Centre for Tropical Medicine and Global Health, University of Oxford, Oxford, United Kingdom; 3 Department of Health Services, Policy, Planning, Management, and Economics, School of Public Health, University for Development Studies, Tamale, Ghana; 4 Liverpool School of Tropical Medicine, Liverpool, United Kingdom; Lahore Medical and Dental College, PAKISTAN

## Abstract

**Background:**

Community pharmacies are the first point of contact for most people seeking treatment for minor illnesses globally. In recent years, the role of community pharmacists has evolved, and they play a significant role in the delivery of public health interventions (PHIs) aimed at health promotion and prevention such as smoking cessation services, weight management services, HIV prevention, and vaccination. This review aims to explore the evidence on the factors that influence community pharmacists to take up the role of delivery of such interventions.

**Methods:**

Three electronic databases namely, Embase (1947-December 2023), Medline (1975-December 2023), and Scopus (1823-December 2023) were searched for relevant literature from the inception of the database to December 2023. Reference lists of included articles were also searched for relevant articles. A total of 22 articles were included in the review based on our inclusion and exclusion criteria. The data were analyzed and synthesized using a thematic approach to identify the factors that influence the community pharmacist’s decision to take up the role of PHI delivery. Reporting of the findings was done according to the PRISMA checklist.

**Findings:**

The search identified 10,927 articles of which 22 were included in the review. The main factors that drive the delivery of PHIs by community pharmacists were identified as; training and continuous education, remuneration and collaboration with other healthcare professionals. Other factors included structural and workflow adjustments and support from the government and regulatory bodies.

**Conclusions:**

Evidence from this review indicates that the decision to expand the scope of practice of community pharmacists is influenced by various factors. Incorporating these factors into the design of policies and public health programs is critical for the successful integration of community pharmacists in the delivery of broader public health to meet the rising demand for health care across health systems.

## Introduction

Community pharmacies are the first point of contact with the health system for most people seeking treatment for minor illnesses globally [1–3]. They are easily accessible, widely distributed, provide quicker services, open for longer hours, and are relatively cheaper than other private health facilities [4–6]. Additionally, they provide a more casual setting for individuals by offering services over the counter for those who do not wish to seek health services from health facilities [7].

The traditional role of community pharmacists has mainly been product-oriented. This entails dispensing prescription and non-prescription medicine, however, this role has evolved to include the provision of various Public Health Interventions (PHIs) due to the increased health demands of the population [1]. This evolution has been endorsed by the International Pharmaceutical Federation (FIP) and is recognized in several income high-income countries such as Australia, the United States, United Kingdom where it has been integrated into existing healthcare models [8]. Low and middle-income countries (LMICs) on the other hand are also recognizing the contribution of community pharmacists to public health however, this role is not well integrated into the broader health system.

Public health entails three main domains; health improvement, health protection, and health service delivery and community pharmacists provide PHIs which contribute greatly to all three domains [9]. These interventions include smoking cessation services such as Nicotine Replacement Therapy (NRT) and counselling services [10]; provision of interventions aimed at promoting health and well-being through changing lifestyle habits, healthy weight management services, advice on healthy living, and participation in health promotion campaigns [11, 12]. In regards to health protection, community pharmacists offer disease control measures, screening for risk factors for non-communicable diseases such as Cardiovascular Disease (CVD) [13, 14], Sexually Transmitted Infections (STIs) screening [15], Human Immune deficiency Virus (HIV) screening [16], provision of immunization services and communicating information on threats to health to patients and the public in general [9]. Health service quality entails the provision of innovative quality pharmacy services to improve health outcomes for instance through medication therapy management services and supporting the safe and effective use of medicine [9]. This review mainly focuses on public health interventions aimed at promoting health and preventing disease as the community pharmacists’ roles are clearly defined in literature.

The delivery of PHIs through community pharmacies not only leads to improved health outcomes but also reduces health inequalities. This is because they are accessible to individuals who lack the resources to access conventional healthcare providers [17]. It also reduces the burden on the health system in two folds; first, it reduces the burden on healthcare providers in facilities with a shortage of healthcare workers [18]. Second, by provision of vaccine and screening services, it reduces the burden of preventable disease in the health care system [19]. Furthermore, the provision of these interventions through community pharmacies leads to reduction of medical treatment costs which leads to savings in healthcare costs [20].

Despite the evidence of such benefits, there is a gap in knowledge on the factors driving community pharmacists to take up this role. Understanding the factors that influence their decision to take up the role is essential for the design of policies in a manner that aligns with their incentives and for the successful implementation of PHI programs. This review therefore aims to explore the factors that influence the community pharmacist’s decision to take up the extended role of PHI delivery.

### Research question

What are the factors that influence community pharmacists’ decision to take up the role of PHI delivery?

### Objectives

The objective of this study was to systematically review available global evidence on the factors that influence community pharmacists’ decision to take up the role of delivery of Public Health Interventions.

## Methods

A protocol for our review can be found in the Open Science Framework [21]. We conducted this systematic review according to the Preferred Reporting Items for Systematic Reviews and Meta-Analyses (PRISMA) Statement [22] and adhered to the PRISMA checklist **S1 Checklist.**

### Search strategy

We searched the literature from July-December 2023 in 3 databases namely: Embase (1947-December 2023), Scopus (18230December 2023), and Medline (1975-December 2023) to identify relevant literature. Both Medical Subject Heading (Mesh) and keywords, Boolean and proximity (e.g. adj2) operators, truncations (*) were used in the search. The search terms used were: “Community Pharmacy” “Private pharmacy” OR “Retail pharmacy” AND “preventive health services” OR “public health services” OR “health promotion” OR “screening” OR “testing” OR “Case finding” AND “cardiovascular disease” OR “Diabetes” OR “Blood pressure” OR “drug use” OR “substance use” OR “mental health” OR “sexual health” OR “vaccination” OR “Immunization” OR “Family planning” OR “Contraception”. This search strategy and terms were modified for Embase and Scopus as appropriate. The search strategy was discussed with the librarian for inclusion sensitivity. Detailed search strategy can be found in the supplementary information **S1 Table.**

We also searched for relevant literature from reference lists of identified studies. The search results from each database were uploaded to End Note 2.0 reference software and duplicates were removed.

### Study selection

Studies were eligible for inclusion if they: 1) reported on interventions delivered in community pharmacies, which are also referred to as private or retail pharmacies. Community pharmacies refer to generally small to medium-sized businesses providing typical pharmacy services such as filling of prescriptions, over-the-counter products, and point-of-care (POC) testing or self-testing kits for common diseases. We included studies conducted in both chain and independent pharmacies. 2) reported on factors that influence the uptake of the delivery of PHIs. 3) interventions were provided by registered pharmacists and/or pharmacy technicians (in some cases referred to as pharmacy assistants); 4) were published in the English language.

Studies were excluded if they: 1) reported on interventions delivered in pharmacies in hospitals, clinics and online pharmacies; 2) reported on interventions aimed at antimicrobial resistance as this is beyond the scope of the broader study, improving treatment and management of diseases, self-medication or management interventions without screening or diagnosis components; 3) Book chapters, reviews, commentaries, letters to the editor, and conference papers.

### Data screening, extraction and analysis

Titles and abstracts were screened by AM in two steps. First, following the removal of duplicates, titles and abstracts were screened against the inclusion and exclusion criteria. Studies that did not meet the criteria were deemed to be irrelevant and excluded. Second, the full articles of the potentially relevant studies were retrieved, and a detailed screening was conducted based on the inclusion and exclusion criteria.

Relevant data from the selected articles were extracted into Ms. Excel by AM with accuracy checks performed on selected articles by JN and any conflict was resolved through discussion of the justification of the inclusion and exclusion criteria. We extracted information on the study title, first author, year of study, study country, PHI and factors influencing the uptake of PHI delivery.

The coding process was conducted manually in MS Excel. Data were analyzed using thematic analysis which entailed 4 phases [23]. Phase 1: familiarization with the identified articles through reading and re-reading, Phase 2: generation of initial codes that were used to develop the coding framework, Phase 3: coding the contents of the articles onto the coding framework, and Phase 4: generation of themes by identifying patterns and relationships across the identified codes. We used established themes to summarize the findings descriptively and summary tables.

### Quality assessment

The studies were assessed for quality independently by AM using the Critical Appraisal Skills Programme (CASP) which uses a standardized checklist to assess the adequacy, trustworthiness, and relevance of the evidence reported in the articles [24, 25]. The CASP checklist evaluates articles on methodological quality, participant recruitment, data collection and analysis, bias, ethical considerations, and the value of the research. For each of the studies a score of 1 (Response of Yes) or 0 (response of No/Can’t tell) was assigned to each of the items assessed, with a maximum score of 10. We classified studies as high (8–10) moderate (6–8) or low (4–6). Studies that scored 6 and above were included in the review. (See S1 Table for more information). The appraisal tool for Cross-Sectional Studies (AXIS tool) to assess for quality of cross-sectional studies [26]. AXIS task contains 20 Yes, No/Somewhat questions to assess the aims, methods, results, and conclusion reported in each study. A score greater than 75% is considered to be high quality, a score of 60%-70% is considered to be of moderate quality and a score less than 60% is considered to be of low quality. The information on quality assessment is in the supplementary files **S1 File.**

## Results

**Fig 1:**
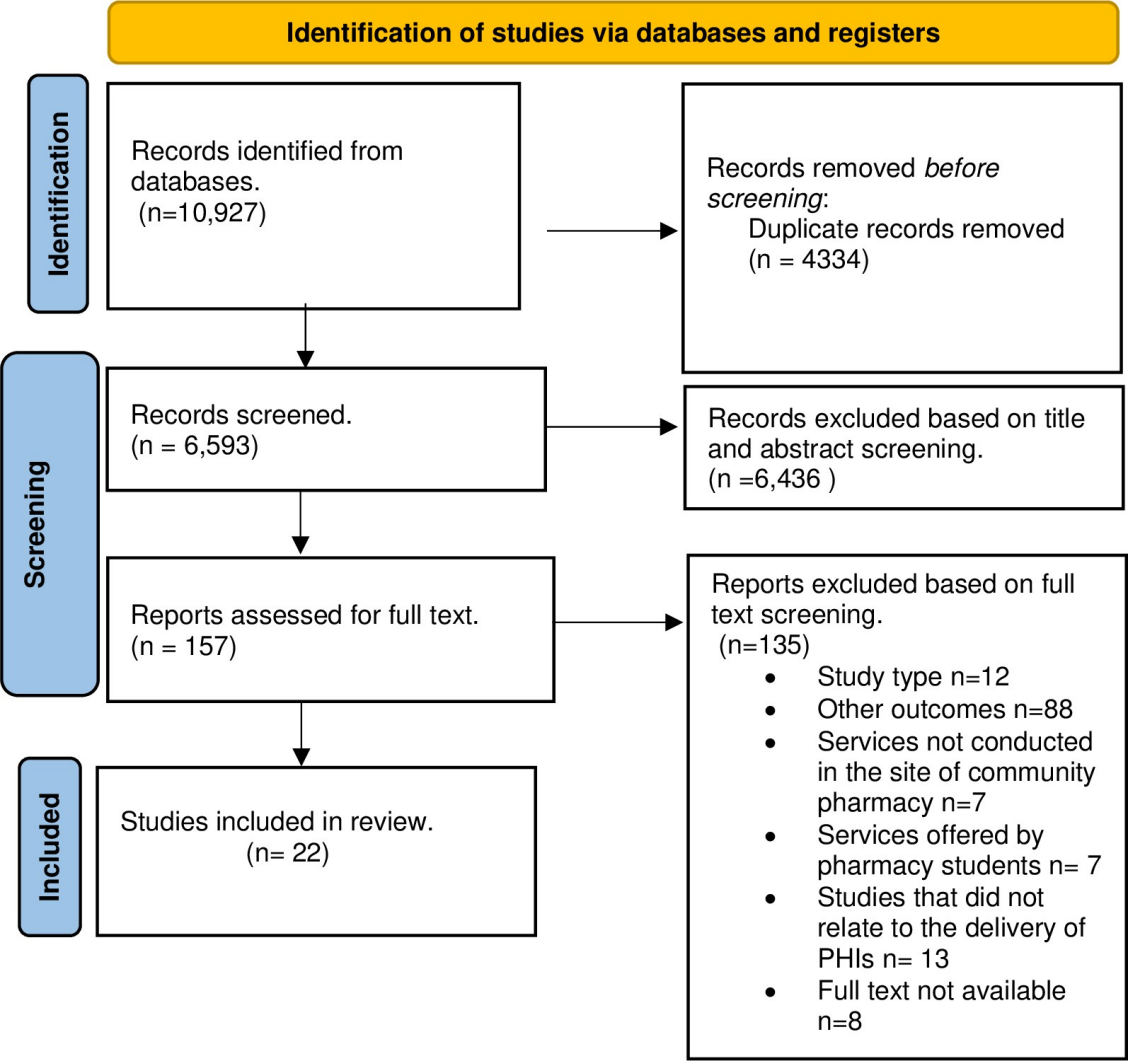
PRISMA flowchart diagram.

Our search yielded 10,927 articles from the three databases, of these 4,434 were duplicates, 6,436 were excluded after the screening of the title and abstracts,157 articles were included in the full-text review, and we included 22 studies in the final review. This selection process is demonstrated in Fig 1.

## Characteristics of the selected studies

Table 1 shows the characteristics of the studies included in the review. Twenty-two studies were selected for the review. Out of the 22, 4 studies were conducted in United Kingdom (UK), 2 in United States of America (USA), 3 in Australia, 4 in Canada, and 1 from Nigeria, Poland, Portugal, Austria, Lebanon, Malaysia, Pakistan, United Arab Emirates (UAE) and Qatar. The main public health interventions identified from the review were; vaccination services (6), healthy weight management (4), Emergency hormonal Contraceptives (EHC) (4), chlamydia screening (2), Cardiovascular screening (CVS) (2), HIV services (2), Health education (1), Pre-exposure prophylaxis screening (PrEP) (1), Diabetes screening (1).

**Table 1:** Characteristics of selected papers.

First Author	Country	Study title	Intervention (PHI)	Identified factors
L. Ahmaro [27]	UK	Investigating community pharmacists’ perceptions of delivering chlamydia screening to young people: a qualitative study using normalisation process theory to understand professional practice	Chlamydia Screening	Training and continuous education
H. Alzubaidi [28]	UAE	Pharmacists’ experiences and views on providing screening services: An international comparison	CVS risk screening	Renumeration. Structural adjustments
Moore S [29]	UK	Health promotion in the high street: a study of community pharmacy	Health promotion activities	Training and continuous education Remuneration Organizational adjustments
M. Atif [30]	Pakistan	A qualitative study to explore the role of pharmacists in healthy weight management in adults in Pakistan: Current scenario and future perspectives	Healthy weight management	Training and continuous education Support from government and Professional bodies
M. Cerbin-Koczorowska [31]	Poland	Is there a time and place for health education in chain pharmacies? Perspectives of Polish community pharmacists	Health education	Training and continuous education Structural and organizational adjustments Remuneration
B. Chirewa [32]	United Kingdom	Emergency hormonal contraceptive service provision via community pharmacies in the UK: a systematic review of pharmacists’ and young women’s views, perspectives and experiences.	Emergency Hormonal Contraceptive	Training and continuous education Collaboration with health care professionals
L. S. Deeks [33]	Australia	Can pharmacy assistants play a greater role in public health programs in community pharmacies? Lessons from a chlamydia screening study in Canberra, Australia	Chlamydia Screening	Training and continuous education Remuneration
S. Almukdad [34]	Qatar	Exploring the role of community pharmacists in obesity and weight management in Qatar: A mixed-methods study.	Weight management services	Structural adjustments Training and continuous education Collaboration with health care professionals
W. C. Ang [35]	Malaysia	Readiness and willingness of Malaysian community pharmacists in providing vaccination services	Vaccination services	Training and continuous education Collaboration with health care professionals Structural and organizational adjustments
L. Chen [36]	USA	Implementation of hormonal contraceptive furnishing in San Francisco community pharmacies.	Contraceptives	Structural and organizational adjustments
M. Donald [37]	Canada	Patient, family physician and community pharmacist perspectives on expanded pharmacy scope of practice: a qualitative study	CVD	Training and continuous education Support from professional organization.
N. Edwards [38]	Canada	Pharmacists as immunizers: a survey of community pharmacists’ willingness to administer adult immunizations	Immunizations	Training and continuous education
I. Figueira [39]	Portugal	Point-of-care HIV and hepatitis screening in community pharmacies: a quantitative and qualitative study	Screening for HIV, Hepatitis C & Hepatitis B	Training and continuous education
R. Hopkins [40]	USA	Support and perceived barriers to implementing pre-exposure prophylaxis screening and dispensing in pharmacies: Examining concordance between pharmacy technicians and pharmacists	Prep screening & dispensing	Training and continuous education Structural adjustment Remuneration
J. E. Isenor [41]	Canada	Pharmacists’ immunization experiences, beliefs, and attitudes in New Brunswick, Canada.	Immunization	Training and continuous education Structural adjustment Remuneration
D. V. Kelly [42]	Canada	Expanding access to HIV testing through Canadian community pharmacies: findings from the APPROACH study	HIV POCT	Training and continuous education Organizational adjustment Remuneration Collaboration with other health care professional
N. Lindne [43]	Austria	The willingness of community pharmacists to immunise: A national cross-sectional study	Immunization	Training and continuous education Support from government
K. P. Osemene [44]	Nigeria	Evaluation of community pharmacists’ involvement in public health activities in Nigeria.	Public health interventions (broad)	Training and continuous education Structural adjustment Collaboration with health care professionals
A. H. Y. Siu [45]	Australia	Implementation of diabetes screening in community pharmacy—factors influencing successful implementation	Diabetes screening	Training and continuous education Remuneration
I. S. Um [46]	Australia	Weight management in community pharmacy: What do the experts think?	Weight management	Training and continuous education Collaboration with health care professionals Structural adjustments
A. E. Weidmann [47]	Scotland	Promoting weight management services in community pharmacy: perspectives of the pharmacy team in Scotland	Weight management	Training and continuous education
D. Youssef [48]	Lebanon	Exploring determinants of community pharmacist-led influenza vaccination in a Middle Eastern country: a national web-based cross-sectional study.	Immunization	Remuneration Support from government

### Quality of evidence

All the included studies had clear statements on the aims, methods used, research design and data collection procedures, data analysis and made significant contributions to existing knowledge and discussed the transferability of the findings to other contexts. However, the relationship between the researchers and participants was not adequately described in 14/16 articles. It was therefore difficult to determine how this relationship may have impacted the findings. For cross-sectional studies assessed using the AXIS tool, the studies met most of the criteria on the tool. However, 5/6 of the studies did not report on measures taken to address non-response bias.

Despite this, we decided to include all the studies in the review as they adequately met the inclusion criteria and contributed to our review objective. (The full details are in the S2 and S3 Tables in S1 File).

### Synthesis of results

We identified 6 major themes from this review: training and education (19/22), structural and organizational adjustments (11/22), remuneration (9/22), collaboration with other health care professionals (6/22), support from government and professional bodies (4/22).

### Training and continuous education

Pharmacists are qualified to provide health care services, however, the reviewed literature suggests that they feel incompetent in taking up this role and would benefit from additional training on the provision of specific PHIs [27, 29–35, 37–47]. Pharmacists in the UK received training on how to deliver chlamydia testing by attending a sexual health learning training session and through the completion of an online learning module [27]. Elsewhere, Moore S et al found that 70% (21/30) of pharmacists acquired health promotion training in their undergraduate degree and informally through reading articles in professional journals rather than attending a formal training program [29]. In Canada, vaccination training was provided to pharmacists through online learning modules and live day training [41]. Almukdad et al, exploring the attitudes of pharmacists in the role of offering weight management services found that pharmacists would benefit from regular and structural training to promote their expertise [34]. Pharmacists in Malaysia reported they would prefer to have at least 1 or 2 days of short training workshops [35].

Pharmacists in Pakistan and Poland expressed a gap in formal training in the undergraduate curriculum and they recommended the inclusion of more practical training sessions in the curriculum [30, 31]. They also expressed concern about the high fees of the courses, and they suggested the need for funding opportunities for free courses. Community pharmacists in Australia and Malaysia reported willingness to provide vaccination services after having received additional short training that covers aspects of needle size gauge and landmarking [41], vaccine storage, technique, and handling of emergency cases [35], with a 2 year renewal interval [43]. Training was suggested for both pharmacists and their assistants in 4 studies to facilitate task shifting of responsibilities [29, 33, 41, 44].

### Remuneration

The provision of PHIs was viewed as an additional workload on top of the dispensing and pharmaceutical-based services and remuneration is a pre-requisite for further activity [28, 29, 31, 33–35, 40, 41]. Pharmacists in Poland expressed they would like to be compensated through an increase in salary to motivate them to take up the role of a health educator [31]. Elsewhere, pharmacists in the UK reported they would prefer to be compensated through a fee-for-service model by the Kingston & Richmond Family Health Service Authority (FHSA) [29]. Pharmacy assistants in Australia viewed remuneration as a tangible recognition for the provision of Chlamydia screening and participation in the training program, in addition to receiving a certificate [33]. None of the other studies reported on the modes of remuneration but there was consensus that remuneration was a means of motivating pharmacists. For instance, pharmacists in the UAE reported that remuneration would motivate them to screen more clients for STIs [28]. There was also an agreement among pharmacists in Australia and the UAE that screening services should be offered at minimal or no costs, funded through government subsidies [28].

### Structural and workflow adjustments

Structural adjustments such as the establishment of a dedicated space for private consultation and as an enabler for the adoption of this role. This was indicated in 8 studies; [29–31, 34–36, 39, 44]. For example, community pharmacists in Nigeria reported that having a designated space for offering patient counseling would encourage them to participate in more public health services [44], similar to findings by Almukdad et al where pharmacists proposed having a private area for counseling as an internal strategy to improve the provision of weight management services (WMS) [34]. The need for a designated space with proper equipment to measure obesity-related parameters for screening blood pressure, weight measurement and cholesterol measurement was reported in Pakistan [30]. For provision of vaccination services pharmacists expressed the need for a specified area for provision of the vaccine services and storage of vaccines under the right temperatures [35, 41]. However, none of the studies detailed how this could be achieved.

Elsewhere organizational adjustments such as having a time dedicated time for delivery of PHIs to avoid disruption of the normal dispensing activities [29, 31, 35, 36, 40] was reported as an enabler for uptake of PHI delivery. Providing training to pharmacy assistants on how to handle pharmacy-based activities was reported as a strategy to free up time for pharmacists to be involved in the delivery of PHIs [31, 44], as well as scheduling appointments for the PHIs [36].

### Collaboration with other healthcare professionals

Pharmacists expressed that the provision of additional services (i.e. delivery of PHIs) would require a multidisciplinary approach [29, 30, 34, 37, 46], especially for cases that required referral and consultation with other health care professionals such as obesity cases [46]. Pharmacists reported they would prefer having multi-disciplinary training courses as a way of forming links between different professional groups [29]. Pharmacists in Canada expressed low awareness among general physicians on pharmacists’ capabilities in providing PHIS and suggested having a shared electronic medical record with physician to facilitate an integrated model of care [37]. Elsewhere in Qatar, pharmacists viewed offering Weight Management Services as complex and they highlighted the need for collaboration with dieticians and physicians for referral purposes [34]. However, the findings from these studies were mainly aspirational, and none reported on an existing collaboration model.

### Support from the government and professional bodies

Support from governing bodies was identified as crucial in the implementation of public health programs in four studies [30, 34, 35, 37]. For example, in Pakistan pharmacists reported there was low awareness of the role pharmacists play in public health and the government could play a role in promoting awareness to the public which in turn would enhance trust from the public [30]. These findings were similar in Canada where pharmacists expressed that professional associations could play a role in creating awareness of this role [37]. Pharmacists in Qatar expressed the need for the Ministry of Health to establish guidelines for pharmacists to facilitate the adoption of Weight Management Services (WMS) [34]. Elsewhere in Malaysia, pharmacists expressed that the government could support them in this role by offering free training and resources to facilitate the vaccination role and professional bodies by advocating for the roles of CPs to be included in vaccination programs [35].

## Discussion

To the best of our knowledge, this is the first review highlighting the factors that influence community pharmacists to expand their scope of practice and deliver PHIs from a global perspective. The findings from this review reaffirm that pharmacists are willing to expand their practice beyond dispensing and take up the role of PHI delivery.

The core finding of this review was that offering additional training to community pharmacists on the delivery of specific PHIs is a requirement to boost the uptake of this role. Training could augment pharmacists’ knowledge and skills as well as empower them to be more competent and confident in the delivery of PHI. The benefits of training pharmacists on PHI delivery include elevating their confidence and competence in service delivery and thus improved health outcomes [49, 50] For instance community pharmacists who received training on provision of smoking cessation services in Thailand reported feeling more confident in offering such services in the future [51, 52]. Similarly, community pharmacists in Australia reported feeling confident in screening clients for risk of cardiovascular disease after receiving training in their pharmacies [53]. Policymakers should therefore put efforts to ensure that community pharmacy providers are qualified and have access to regular training. There was no reported standardized model in place for training pharmacists on PHI delivery from the studies reviewed. Nevertheless, various training models have been adopted elsewhere although these differ by context and the PHI under consideration [54–57].

These training models include peer learning, which has the potential to influence the practitioner’s behaviour [54], learning at work [55, 56], and formal certification to become specialists [57]. These trainings are delivered through different formats such as face-to-face learning (onsite/off-site), and online learning (webinars-learning modules and activities). Face-to-face training has been reported as the preferred mode of delivery by pharmacists in Ethiopia [55], UAE [58], and USA [59], as it offers an opportunity for quick feedback from the instructor, and provides an opportunity for peer networking, which could enhance collaboration with healthcare professionals in other fields. Online training on the other hand has been reported as a preferred mode of delivery by pharmacists in Australia [60], as it offers the convenience of pharmacists completing modules at their own pace and schedule. Blending diverse training methods and modes by pharmacists’ preferences is key to ensuring that pharmacists are well-equipped to take up the role of delivery of PHI. Additionally, modifications of the undergraduate curriculum to include public health modules is a starting point to improving professional skills and perceptions of pharmacists towards taking up this extended role [61, 62].

Government support to community pharmacies is crucial in enhancing the uptake of PHI delivery. This can be through the provision of resources to facilitate the adoption of PHI delivery through, for example, the provision of materials to promote awareness and offering equipment at a subsidized price that is affordable to pharmacists [63]. The vital role that community pharmacies play in improving health indicators is recognized globally, however, their inclusion in the design of policies, countries’ health strategies, regulations and monitoring is minimal [64]. The government can therefore play a role by establishing clear guidelines, policies, and regulatory frameworks to guide the integration of this role into broader health systems tailored to the unique landscape of each country. For instance, in Saudi Arabia, MoH has developed specific guidelines on the provision of immunization services within community pharmacies [65]. Elsewhere in Kenya, MOH through the National AIDS and STI Control Programme (NASCOP) launched guidelines which advocated for the delivery of HIV-Self test kits in community pharmacies [66]. Furthermore, the government and professional bodies could promote national campaigns to create awareness of the crucial role that pharmacists play in public health.

Whilst integrating community pharmacist PHI delivery role into the broader health system, it’s important that they generally operate independently of other healthcare providers in a retail environment [67]. Therefore, training sessions could create an avenue to foster interprofessional collaborations between pharmacists and other healthcare professionals.

Interprofessional collaboration between physicians and pharmacists has resulted in improved patient outcomes and a reduction of health system inefficiencies and costs [68, 69]. This has led to the establishment of Collaborative Practice Agreements (CPAs) in the USA where community pharmacists conduct screening for chronic infections in the pharmacies and hand off the reactive cases to general physicians. This helps to close the gap of loss to follow-up patients [70]. Elsewhere in UK general physicians have communication channels for referral of patients for a same day consultation with community pharmacists and vice versa [71]. Interprofessional collaboration has been described as an evolving process that progresses through a series of stages described by various collaboration models. For instance, a GP- pharmacist model by McDonough and Doucette [72] describes it as a progression in 4 main stages: stage 0- professional awareness, stage 1- professional recognition, stage 2- exploration and trial stage, stage 3- professional relationship expansion, stage 4- a commitment to the collaborative working relationship and is influenced by different factors such as proximity, time, clinical knowledge, communication, mutual interests and professional equality. Other models have been adopted for collaboration between pharmacists and GPs and are similar in that collaboration progresses from brief interactions to a clearly defined relationship where the roles of both cadres are well defined [73, 74]. Role clarity has been shown to influence the adoption of role expectations and task performance [75, 76].

Community pharmacies are private retail businesses operating within a competitive market and aim to maximize profits to survive in the market, it is therefore not surprising that remuneration influences the uptake of the additional role. Delivery of PHI is viewed as an additional role and pharmacists have few incentives to deliver the expanded services if the compensation is inadequate. Although the remuneration of community pharmacists has mainly been based on their retailing and dispensing functions, a few countries have introduced payment mechanism reforms as a means of encouraging the adoption of this role [77]. For instance, the fee-for-service model has been adopted to encourage pharmacists to provide smoking cessation services [78], influenza vaccination [79], and diabetes-related education, training and monitoring in the community settings [80] and was more preferred by pharmacists as it was easy to implement and integrate into the existing business model [81]. Pay-for-performance model has been used in a UK program, where pharmacies were renumerated based on the number of people who successfully quit smoking [82]. However, there is a gap in knowledge on the preferred payment model in various contexts. Understanding the payment model preferences of community pharmacists is a crucial knowledge gap as it has major implications on the implementation, adoption, and potential impact of pharmacists’ payment model.

Finally, structural and workflow adjustments such as having a designated space and having a dedicated time play a role in community pharmacists taking up the role of PHI. The importance of a private room has been stressed in several studies as a way of building trust and maintaining confidentiality for patients who want to discuss sensitive medical issues such as requests for EHC, screening for STIs, Prep, and HIV screening [83, 84]. Evidence suggests that community pharmacists have a preference for having a private consultation room to provide services for diabetes management to preserve patients’ privacy and confidentiality [85]. This can be achieved through the establishment by development of policies and standards for the physical space of pharmacies such as having a designated space for conducting PHIs. For instance, having a private space has been incorporated as a requirement in Western Australia section 7 of Pharmacy Regulations 2010, which specifies that, “*The premises are to have an area in which a consultation conducted by a pharmacist is not reasonably likely to be overheard by a person not a party to the consultation”* [86]. These guidelines are backed up by the professional body code of ethics as a means to ensure that the client’s right to privacy and confidentiality is maintained [87]. Workflow adjustments such as having time dedicated to PHIs would allow pharmacists to plan efficiently and allocate sufficient time to deliver high-quality services. However, evidence shows that community pharmacists have a general preference for being easily accessible to patients by taking walk-in clients [85].

### Limitations

One limitation of this review was that most of the findings from were mainly aspirational and therefore minimal data on various mechanisms that have been applied to facilitate the role of PHI delivery by community pharmacists, however, this will be addressed by a broader study. Second, the findings from this review were mainly in studies conducted in high-income countries and therefore the findings may not be contextually replicable in low-income settings as factors vary across different contexts. To overcome these limitations, further empirical work in LMIC settings is required to determine the key drivers, policy, and practical considerations for delivery of PHIs. Finally, the studies included in this review were those published in English language and there is a likelihood that we might have missed some articles that may be relevant to our review. Despite these limitations, this manuscript provides crucial information that has great potential to inform the design of public health policies targeting community pharmacists.

### Study implications

This review highlights the different factors that play a key role in influencing community pharmacists’ decision to take up the role of PHI delivery. However further research is needed to generate evidence on how these factors interact to influence implementation practices and sustainability. This research could entail identifying context specific barriers and facilitators of PHI delivery in community pharmacies particularly in LMICs. This information could inform the design of implementation strategies that can enhance sustainability of PHI programs adopted in community pharmacies. Such research is crucial for two reasons: first, it will ensure that policies are designed in a manner that incentivizes community pharmacists to take up this role. Second, it will facilitate the establishment of guidelines to standardize community pharmacy practice and integration of this role into broader health systems which will in turn enhance the contribution of community pharmacists to public health.

## Conclusion

This review sheds light on the various factors that influence the decision of community pharmacists to expand the scope of practice and take up the role of delivery of public health interventions. Incorporating these factors into the design of policies and public health programs is crucial for the successful integration of community pharmacists into broader public health initiatives. However, these findings do not indicate the relative importance that is placed on each of the factors by community pharmacists. The findings from this review will inform the design of a discrete choice experiment to elicit context-specific preferences of community pharmacists for the identified factors, which will in turn contribute to the design of policies that will enhance the contribution of community pharmacists to public health.

## Supporting information

S1 Checklist PRISMA 2020 checklist. (DOCX)

S1 Table Search strategy. (DOCX)

S1 File Quality assessment findings. (DOCX)
