# Supplementary material for: Factors influencing the uptake of public health interventions delivery by community pharmacists: A systematic review of global evidence
Source: PLoS One. 2024 Aug 1;19(8):e0298713. doi: 10.1371/journal.pone.0298713 (PMC11293714; doi:10.1371/journal.pone.0298713)
Supplement: S1 Table — (DOCX) [file pone.0298713.s002.docx]

**Database: Ovid MEDLINE(R) ALL <1946 to December 14, 2023>**
**Search Strategy:**
**1**  exp "pharmacy (shop)"/ (0)
**2**  ((retail or private or communit* or commercial) adj pharmac*).mp. [mp=title, book title, abstract, original title, name of substance word, subject heading word, floating sub-heading word, keyword heading word, organism supplementary concept word, protocol supplementary concept word, rare disease supplementary concept word, unique identifier, synonyms, population supplementary concept word, anatomy supplementary concept word] (13225)
**3**  1 or 2 (13225)
**4**  (screen* or test* or case finding).mp. [mp=title, book title, abstract, original title, name of substance word, subject heading word, floating sub-heading word, keyword heading word, organism supplementary concept word, protocol supplementary concept word, rare disease supplementary concept word, unique identifier, synonyms, population supplementary concept word, anatomy supplementary concept word] (5590941)
**5**  (cardio-vascular or cardiovascular or diabetes or "blood sugar" or hypertension or "blood pressure" or cholesterol or lipid or heart or coronary or kidney or renal or osteoporosis or cancer or asthma or dental or "oral health" or eye or ophthalmic or obesity or injur* or smoking or alcohol or "drug? *use*" or "substance? *use*" or "mental health" or sexual* or pregnancy or febrile or fever? or hepati* or influenza or flu or covid-19 or coronavirus* or corona-virus* or ebola or hiv or malaria or lice or tuberculosis or respiratory or pulmonary or topical or skin or dermatolog*).mp. [mp=title, book title, abstract, original title, name of substance word, subject heading word, floating sub-heading word, keyword heading word, organism supplementary concept word, protocol supplementary concept word, rare disease supplementary concept word, unique identifier, synonyms, population supplementary concept word, anatomy supplementary concept word] (13712707)
**6**  4 and 5 (2535617)
**7**  (preventive health service or prophyla* or public health service* or health promotion).mp. [mp=title, book title, abstract, original title, name of substance word, subject heading word, floating sub-heading word, keyword heading word, organism supplementary concept word, protocol supplementary concept word, rare disease supplementary concept word, unique identifier, synonyms, population supplementary concept word, anatomy supplementary concept word] (325761)
**8**  (vaccin* or immuni#ation or inoculation).mp. [mp=title, book title, abstract, original title, name of substance word, subject heading word, floating sub-heading word, keyword heading word, organism supplementary concept word, protocol supplementary concept word, rare disease supplementary concept word, unique identifier, synonyms, population supplementary concept word, anatomy supplementary concept word] (637414)
**9**  (contracept* or family planning).mp. [mp=title, book title, abstract, original title, name of substance word, subject heading word, floating sub-heading word, keyword heading word, organism supplementary concept word, protocol supplementary concept word, rare disease supplementary concept word, unique identifier, synonyms, population supplementary concept word, anatomy supplementary concept word] (122690)
**10**  7 or 8 or 9 (1056891)
**11**  6 or 10 (3459989)
**12**  3 and 11 (2534)
