# Supplementary material for: Factors influencing the uptake of public health interventions delivery by community pharmacists: A systematic review of global evidence
Source: PLoS One. 2024 Aug 1;19(8):e0298713. doi: 10.1371/journal.pone.0298713 (PMC11293714; doi:10.1371/journal.pone.0298713)
Supplement: S1 File — (DOCX) [file pone.0298713.s003.docx]

**Table 2: Assessment of qualitative studies**

| Appraisal criteria | Yes | Somewhat | No/Not clear |
| --- | --- | --- | --- |
| 1. Was there a clear statement of the aims of the research? | 16 |  |  |
| 1. Is a qualitative methodology appropriate? | 16 |  |  |
| 1. Was the research design appropriate to address the aims of the research?   Has the researcher justified the research design? | 16 |  |  |
| 1. Is the recruitment strategy appropriate for the study aims?   Researcher explained how the inputs/outputs/study participants were selected?  Discussion around recruitment, i.e., why some inputs/outputs/participants were not chosen? | 14 | 2 |  |
| 1. Does the data collection approach appropriately answer the research question?  - Was the data collection location justified? - If it clear how data were collected? - Were data collection methods clear? | 16 |  |  |
| 1. Has the relationship between the researcher and participants adequately been considered?  - Researcher reflexivity and potential partiality during the formulation of research questions or data collection? | 2 |  | 14 |
| 1. Did the researcher consider ethical issues before conducting the study?  - Are issues on informed consent and confidentiality adequately addressed? - Did the researchers seek ethical approval? | 14 |  | 2 |
| 1. Was there adequate rigor during data analysis?  - An explicit explanation of how the analysis was conducted. - A clear statement of how themes/categories were developed. - Are there proper considerations to inconsistent findings? | 14 | 2 |  |
| 1. Are the findings reported clearly?  - Explicit findings - An adequate discussion of evidence for and against the researcher arguments - The credibility of finds (triangulation, respondent validation, more than 1 analysis) findings are discussed in relation to the original research question | 16 |  |  |
| 1. How valuable is the research?  - The researcher explains how the study contributes new knowledge or adds to existing knowledge. - Have the research identified new areas for future research. - Are there clear explanations about how the findings can be applied to other settings. | 16 |  |  |

**Table 3: Assessment of Cross sectional studies**

| AXIS-Cross sectional studies checklist | Yes | Somewhat | No/Not clear |
| --- | --- | --- | --- |
| Were the aims /objectives of the study clear? | 6 |  |  |
| Was the study design appropriate for the stated aim? | 6 |  |  |
| Was the sample size justified? | 6 |  |  |
| Was the target/reference population clearly defined? (Is it clear who the research was about?) | 6 |  |  |
| Was the sample frame taken from an appropriate population base so that it closely represented the target/reference population under investigation? | 5 | 1 |  |
| Was the selection process likely to select subjects/participants that were representative of the target/reference population under investigation? | 6 |  |  |
| Were measures undertaken to address and categorize non-responders? |  |  | 6 |
| Were the risk factor and outcome variables measured appropriate to the aims of the study? | 6 |  |  |
| Were the risk factor and outcome variables measured correctly using instruments/measurements that had been trialed, piloted or published previously? | 4 | 2 |  |
| Is it clear what was used to determined statistical significance and/or precision estimates? (e.g., p-values, confidence intervals) | 6 |  |  |
| Were the methods (including statistical methods) sufficiently described to enable them to be repeated? | 6 |  |  |
| Were the basic data adequately described? | 6 |  |  |
| Does the response rate raise concerns about non-response bias? |  |  | 6 |
| Were the results internally consistent? | 6 |  |  |
| Were the results presented for all the analyses described in the methods? | 6 |  |  |
| Were the author’' discussions and conclusions justified by the results? | 6 |  |  |
| Were the limitations of the study discussed? | 4 |  | 2 |
| Were there any funding sources or conflicts of interest that may affect the authors’ interpretation of the results? |  |  | 6 |
| Was ethical approval or consent of participants attained? | 6 |  |  |
